# A new method to monitor bone geometry changes at different spatial scales in the longitudinal *in vivo μ*CT studies of mice bones

**DOI:** 10.1371/journal.pone.0219404

**Published:** 2019-07-22

**Authors:** Yang Zhang, Enrico Dall’Ara, Marco Viceconti, Visakan Kadirkamanathan

**Affiliations:** 1 Department of Automatic Control and Systems Engineering, The University of Sheffield, Sheffield, United Kingdom; 2 INSIGNEO Institute for in Silico Medicine, The University of Sheffield, Sheffield, United Kingdom; 3 Department of Oncology & Metabolism, The University of Sheffield, Sheffield, United Kingdom; 4 Department of Industrial Engineering, Alma Mater Studiorum, University of Bologna, Bologna Area, Italy; 5 Medical Technology Lab, IRCCS Istituto Ortopedico Rizzoli, Bologna, Italy; University of Zaragoza, SPAIN

## Abstract

Longitudinal studies of bone adaptation in mice using *in vivo* micro-computed tomography (*μ*CT) have been commonly used for pre-clinical evaluation of physical and pharmacological interventions. The main advantage of this approach is to use each mouse as its own control, reducing considerably the sample size required by statistical power analysis. To date, multi-scale estimation of bone adaptations become essential since the bone activity that takes place at different scales may be associated with different bone mechanisms. Measures of bone adaptations at different time scales have been attempted in a previous study. This paper extends quantification of bone activity at different spatial scales with a proposition of a novel framework. The method involves applying level-set method (LSM) to track the geometric changes from the longitudinal *in vivo μ*CT scans of mice tibia. Bone low- and high-spatial frequency patterns are then estimated using multi-resolution analysis. The accuracy of the framework is quantified by applying it to two times separated scanned images with synthetically manipulated global and/or local activity. The Root Mean Square Deviation (RMSD) was approximately 1.5 voxels or 0.7 voxels for the global low-spatial frequency or local high-spatial frequency changes, respectively. The framework is further applied to the study of bone changes in longitudinal datasets of wild-type mice tibiae over time and space. The results demonstrate the ability for the spatio-temporal quantification and visualisation of bone activity at different spatial scales in longitudinal studies thus providing further insight into bone adaptation mechanisms.

## Introduction

Bone has a dynamic structure that is modified through bone modelling and remodelling. Bone is shaped or reshaped through the spatio-temporal modelling process where the bone formation and resorption take place independently [1]. Meanwhile, bone undergoes the remodelling process at discrete bone sites which maintains the skeletal strength through the coupled bone resorption and formation [2–4]. Musculoskeletal disease are usually associated to imbalance in bone remodelling [4]. To better investigate these skeletal diseases, mouse models have been widely used in preclinical studies for reasons of cost- and time-efficiency. Mouse models are routinely used to investigate the effect of interventions against musculoskeletal diseases. To evaluate the effects, it is essential to be able to monitor and quantify bone activities.

Traditionally, bone activity can be measured from two-dimensional (2D) histological slides using dynamic histomorphometry methods [5, 6]. Biochemical markers of bone formation are injected into a bone biopsy twice in time-separated sequences to fluorochrome label the mineralized bone surface at different times. The labelled bone formation surface may appear as scalloped or as smoothed reversal lines, identified as remodelling-based and modelling-based bone formation, respectively [6, 7]. The dual fluorochrome labels can therefore be used to assess the properties of bone modelling and remodelling, such as cortical width, mineralizing surface, bone formation rate, etc. [8, 9].

Recently, the development of the micro-computed tomography (*μ*CT) technique enables the 3D monitoring of the skeletal structure of living rodents [10–12]. The measurement is based on the 3D rigid image registration of the time-lapsed *in vivo μ*CT scans. The bone formation and resorption are detected according to the appearance and disappearance of the voxels at consecutive scans [5, 13, 14]. The use of *in vivo μ*CT measurements enables longitudinal studies where each mouse is the control of itself; this can reduce the number of animals required to achieve properly powered conclusion of 60%, if compared to cross-sectional studies [15, 16]. The approach also enables the analysis over whole bones, including both cancellous and cortical bone, providing for the first time an experimental view of how bone adaptation occurs over the anatomical space and over time [15]. This approach has been widely applied for the characterization of bone properties, such as the influence of age on bone adaptation and bone surface mineralization [13, 17], or the longitudinal effect of ovariectomy and Parathyroid Hormone treatment on morphological properties of mouse tibia [16, 18].

*μ*CT scan based methods reveals the complexity of bone dynamic structures and more specifically that bone geometry changes over space and time [7, 18]. The analysis of these spatio-temporal patterns would be of great benefit to get an insight of the bone adaptation. A previous study attempted to classify and quantify bone adaptation in the tibia midshaft from the *μ*CT 3D images according to the time sequence of bone volume changes temporally [7]. This work represents the first attempt at categorising bone adaptations at multiple scales over time. The changes at bone surfaces were classified into eight categories—inactive, short/long term formation, short/long term resorption, remodelling, fast remodelling and mixed sequences of formation followed by resorption according to the different combination of resorption, formation and quiescent over time at each voxel.

Methods to analyse bone adaptation across spatial scales are limited. The *μ*CT morphometric analysis of trabecular and cortical bone in small rodents with *μ*CT was found to be highly reproducible [12, 19] and was used to assess longitudinal changes in bone structure by integrating automatic segmentation and rigid registration [20–22]. Waarsing *et al*. [23] developed a prototype to detect and track local bone structure changes over time through the registration of the *in vivo μ*CT images of the same bone of the same animal at different time points. Scheulte *et al*. [5] further extended this approach to visualise and quantify bone formation and resorption parameters over time and it was successfully applied to analyse the effects of cyclic mechanical loading on mouse tail vertebrae [24]. Nevertheless, all these studies were performed on rodents, that present bone growth during the largest proportion of life expectancy [12]. The growth, that contributes to the considerable bone structure changes, presents a great challenge for the study of bone adaptation, especially long bones [22]. To minimise the effect of growth, bone morphometric analysis have been usually limited to small diaphysis regions [8, 25, 26]. Lu *et al*. [27, 28] performed the quantification of bone adaptation in larger bone volumes over time by partitioning the volume of interest (VOI) into sub-regions. However, the analysis was restricted to a single spatial scale but it is known that bone adaptation occurs across scales. For example, bone modelling and remodelling take place at different bone regions and can contribute to bone geometry changes at different spatial scales [29, 30]. The observation made in preclinical studies showed that the interventions (biochemical and/or biomechanical stimuli) can also affect bones at different scales [31].

To explore bone activities according to the spatial scales, a multi-resolution framework is proposed in this study. This framework is applied to the cortical bone surface of the same large volume of interest (80% of mouse tibia) and provides high resolution quantification and visualisation of bone adaptation. To the authors’ knowledge it is the first time attempt to estimate bone changes at multiple spatial scales. It is a substantial advancement from the previous study presented by Lu et al. [28]. This study could be further applied to support the interpretation of bone adaptations and the effects of different interventions and treatments. In the proposed framework, the level-set method (LSM), which has the capability to capture complicated topology evolution, is first employed for segmentation of bone structure from the *μ*CT images, and then to quantify the spatio-temporal geometry changes on the bone surface [32]. A multi-resolution analysis is then performed using dual-tree complex wavelet transform (CWT) towards characterising the bone changes at low- or high-spatial frequency. The dual-tree CWT is employed for its properties of near shift invariance and directional selectivity in multi-dimensions [33, 34]. This novel framework is applied to the entire cortical surface of mouse tibia for the quantification and visualisation of bone activities at different spatial frequencies.

The paper is organised as follows: firstly, the materials and methods section describes the *μ*CT imaging and its preprocessing, hypothesis assumed and the multi-resolution algorithm for separation of high- and low-spatial frequency events. Then the results section describes the outcomes of the analysis framework to evaluate the algorithm accuracy, and then it reports the outcomes of the application of the method to study the right tibia of wild-type mice. Finally, a discussion of the results and conclusions are provided.

## Materials and methods

### Animal model

For this study, images from the right tibia of female wild-type C57BL/6J(BL6) mice between week 14 and week 22 of age were used. The detailed information can be found in Lu *et al*. [27]. The procedures were approved by the local Research Ethics Committee of the University of Sheffield (Sheffield, UK).

### Ethics statement

14-week-old female C57BL/6J (BL6) mice were purchased from Harlan Laboratories (Bicester, UK). Prior to the experiment, the mice were allowed to acclimate to the new environment for one week and were housed in the same environmentally controlled conditions with a 12-h light/dark cycle at 22°*C* and had free access to food and water. All the procedures were complied with the UK Animals (Scientific Procedures) Act 1986 and were performed under the project license approved by the UK Home Office (PCF1D350B).

### *In vivo μ*CT scanning and image processing

In this study, three different animal models were used and listed as follows:

- Dataset I: a set of repeated consecutive *in vivo μ*CT scans of the same tibia were used for the evaluation of the algorithmic accuracy (Number of mice: *N* = 5) [27].
- Dataset II: a set of synthetically modified scans based on the first group of dataset were used to evaluate the accuracy of the method under conditions of known global and/or local bone activity (*N* = 5). More details are given in the section of evaluation of the algorithmic accuracy.
- Dataset III: a set of *in vivo μ*CT scans performed weekly on the same female wild-type mouse from week 14 to week 22 of ages, to test the applicability of the method (*N* = 5) [18].

Every tibia was scanned with an *in vivo μ*CT system (Scanco VivaCT80, Switzerland) with the following scanning parameters: voxel size 10.4*μm*, voltage 55*keV*, intensity 145*μA*, field of view 32*mm*, samples/projections 1500/750, and integration time 200*ms*. A third-order polynomial beam hardening correction determined using a 1200*mgHA*/*cm*^3^ wedge phantom was applied during the reconstruction [27, 28].

In the image processing, firstly, the repeated scans were aligned in the same anatomical reference system. The first-time scan was transferred to its anatomical position [27] and was referred as baseline. The rigid registration that used Euclidean distance similarity measurement and Quasi-Newton optimizer was applied to transfer the following scans of the same tibia to the baseline scan.

Secondly, bone length was measured using the minimum bounding box that started from the pixel of the most proximal tibial bone to the most distal bone. The fibula was removed just above the tibiofibular syndesmosis and the region of 80% tibial length starting from the proximal growth plate was reserved as the VOI in this study.

Afterwards, the grayscale images were smoothed with the 3D Gaussian filters with standard deviation of 0.65 and kernel size of [3, 3, 3]. Bone tissues were segmented using the global thresholding and the threshold was automatically chosen using Otsu’s method that selects the threshold by minimising the interclass variance of the tissue (white) and background (black) [35]. The images before and after segmentation were compared in order to evaluate the quality of the automatic thresholding.

### Hypothesis

Bone tissue is a highly porous material, with porosities that vary quite considerably in size. Here we operate with images at voxel size of 10.4*μm*, and we assume that any free surface at that resolution can in principle be a site of new bone formation or of existing bone resorption. While there are small porosities, these are smaller than most cells, so we can safely assume that no biological activity occurs over those free surfaces. In consistency with most literature on the subject, we also assume that bone formation and resorption occur normal to the bone surface [36].

### The framework for exploring spatial frequency of tibial geometry changes

#### Overview

**Fig 1:**
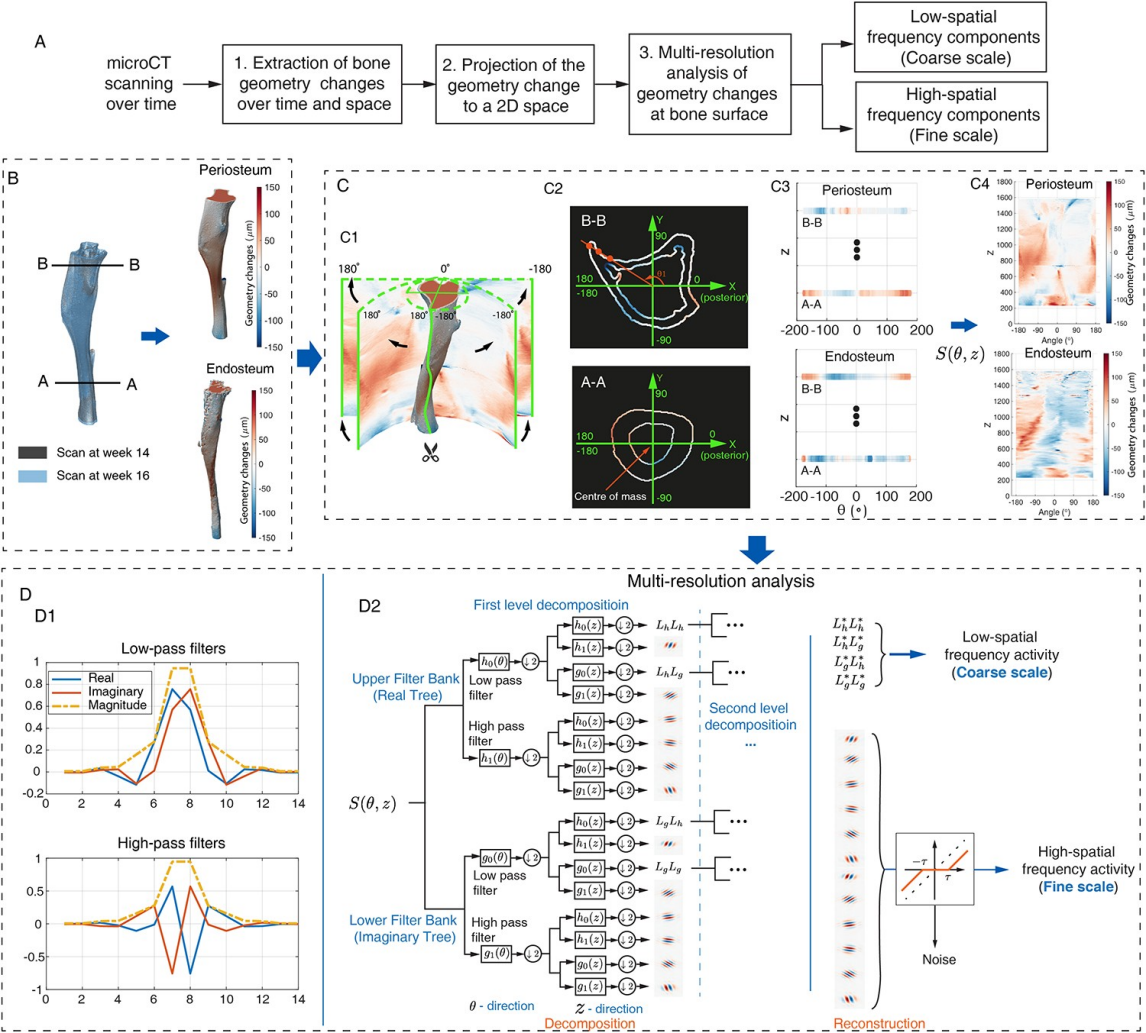
The framework for separating bone activity at different spatial scales. (A) The flowchart of the proposed framework. (B) Extraction of bone geometry changes over space and time. Left: the periosteum at week 14 (surface in gray) and week 16 (surface in blue). Right: extracted geometric changes at periosteum (top) and endosteum (bottom) from week 14 to week 16. (C) Projection of the geometry changes into a 2D space. Sub-figure C1 showed the construction of the 2D space to indicate the geometric changes, where the horizontal dimension corresponds the the angles (°) to tibia posterior (illustrated in sub-figure C2) and the vertical dimension corresponds to bone height (*voxel*). Sub-figure C2 is the segmented periosteum and endosteum of the cross-views *A* − *A* and *B* − *B* (see sub-figure B), where color indicates the geometry changes over time and the green. The corresponding projection was given in sub-figure C3. The constructed 2D maps of periosteum and endosteum corresponding to the sub-figure B were given in sub-figure C4. (D) Multi-resolution analysis of the geometry changes using dual-tree complex wavelet analysis. The corresponding high/low pass filters were designed using q-shift solution of length 14 and shown in sub-figure D1. The dual-tree structure of the filter banks were shown in sub-figure D2. In sub-figures B and C, the colourmap is used to indicate the direction and the amplitude of geometry changes. The warm colour indicates that the bone surface evolves along outward-pointing normal direction (i.e., Periosteum: bone formation; Endosteum: bone resorption) whereas the cold colour indicates the surface evolving in the opposite normal direction.

The analysis of tibial geometry changes according to spatial frequencies is performed through the following steps (Fig 1A):

- Extraction of tibial geometry changes from *μ*CT scanning;
- Description of the 3D geometry changes in a 2D map, *S*;
- Multi-resolution analysis of the 2D map;

#### Extraction of tibial geometry changes over time and space

The LSM was employed to reconstruct the tibia surface after image processing. Specifically, the bone surface is described implicitly by the zero level of a function, Φ_*t*_(Ω, *t*), defined over a spatial region, Ω, and time, *t*,

$$\begin{array}{c} \Gamma (t)=\{\Omega |\Phi (\Omega ,t)=0\} \end{array}$$ (1)

where Γ(*t*) is the set of 3D coordinates of bone surface at time *t*. The level-set function is defined as a signed distance function of the bone surface. The evolution of the bone surface, therefore, can be described by a level-set equation [32],

$$\begin{array}{c} \frac{\partial \Phi }{\partial t}+F\left|\nabla \Phi \right|=0 \end{array}$$ (2)

where *F* is the speed field describing the evolution speed of the bone surface along the normal direction. Since the signed distance function has the properties of |∇Φ| = 1 [32], Eq (2) is discretised as,

$$\begin{array}{c} F_{t}=-(\frac{\Phi_{t+1}-\Phi_{t}}{\Delta t}) \end{array}$$ (3)

where Δ*t* is the time interval between successive observations of bone surfaces. This indicates that the bone surface evolution along the normal direction (i.e., bone geometry changes) can be calculated by the difference in level-set functions at consecutive observation time points.

In this study, the tibia geometry includes both the periosteum and the endosteum. After image processing, the bone surfaces were extracted from the binary images, and it consisted of several closed surfaces. The surface of the largest area was isolated and labelled as periosteum while the remaining surfaces were labelled as the endosteum. To check for the segmentation accuracy, the labelled surfaces were compared to the binary images. This was done by reconstructing the periosteum and the endosteum independently using the LSM, given in Eq (1). The reconstructed surfaces from the level-set function were then compared to the segmentation results obtained using thresholding and the marching cubes method. [37] The Hausdorff distances between these two were found to be below 0.5 voxel, thus confirming their consistency (S1 Fig in the supplementary).

#### Projection of the 3D geometry changes into 2D map

Our multi-resolution analysis was based on a 2D rectangle feature array. To perform this, the tibial geometry changes were unwrapped into a 2D space, defined by the bone height and the angle to the tibia posterior side. The posterior side was determined to be along the positive side of the X-axis in the transverse section (*X* − *Y*) of the tibia in the registered reference system (Fig 1C). The projected 2D map of geometry changes, *S*(*θ*, *z*), was used as the input for the multi-resolution analysis.

Due to the complexity of tibial geometry, some of the cross-views presented the concave shapes may introduce the situation of surjective projection. For example, in the cross-view B-B shown in Fig 1C2, three voxels at periosteum were projected into the same location at the 2D space. In those situations, the voxel with the largest geometry change was selected. Extra errors were therefore introduced and were quantified by an error rate calculated by

$$\begin{array}{c} e_{r}=\frac{N_{mis}}{N_{tot}} \end{array}$$ (4)

where *N*_*tot*_ is the total number of voxels and *N*_*mis*_ is the number of the voxels mapped incorrectly. The error rate at periosteum was 1.8% ± 0.7% and endosteum is 2.8% ± 0.9% (calculated from the scans of five wild-type mouse tibiae over eight weeks). It should be pointed out that such surjective projection issues are rare.

In addition, the influence of the differences in the radius, defined from centre of mass to the segmented bone surface, was not considered. This could lead to a scenario where the same angle in the 2D map corresponds to different arc length at tibia surface. However, the differences of radius at adjacent angles were smaller than half a voxel, and are the corresponding differences at arclength. The fact that wavelet analysis captures the features at local neighbourhood, helps to reduce to a minimum the effect of these differences on the analysis.

#### Multi-resolution analysis of tibial geometry changes

The wavelet transform was introduced to separate a signal in accordance with the frequencies. In this study, the CWT in the dual-tree structure was applied, as shown in Fig 1D. The input signal, *S*(*θ*, *z*), was passed through two separate discrete filter banks that gave the real and imaginary parts of the wavelet transform, i.e., the upper and lower filter banks [33, 38]. At each filter bank, the signal was divided into two frequency bands using a low pass filter and a high pass filter with a down-sampling factor of 2. *h*_0_(*θ*) and *g*_0_(*θ*) are two low-frequency filters while *h*_1_(*θ*) and *g*_1_(*θ*) are the corresponding high-frequency filters. The filters were obtained using the q-shift algorithm of length 14, illustrated in Fig 1D [34, 38]. After the first level decomposition at direction *θ* and *z*, *S*(*θ*, *z*) was decomposed into 4 low-spatial frequency components (coarse scale) and 12 high-spatial frequency components (fine scales) that are associated with the pattern of *S*(*θ*, *z*) at six directions: 15°, 45°, 75°, −75°, −45°, −15°. To investigate *S*(*θ*, *z*) at the coarser scales, the low frequency components were passed through to a second level decomposition, with the same filter banks and the process was repeated recursively through further coarse scales. The decomposition was terminated at the 4th level, where the resolution of the coarse pattern was 0.28 × 166*μm*. The resolution in the *z*-dimension was close to the dimension of a resorption cavity [39].

Subsequently, the low-frequency pattern was synthesised by reversing the decomposition process using the coefficients at the coarsest scale (obtained after the low-pass filter at highest level decomposition). In order to reduce the high frequency noise inherent in the imaging process and possibly introduced in the pre-processing stage, a soft global thresholding was performed on the coefficients at high frequencies:

$$\begin{array}{c} \left\{ \begin{array}{c} \hat{d}=\text{sgn}\left(d\right)\max(|d|-\tau ,0)\;\left|d\right|>\tau \\ \hat{d}=0\;\left|d\right|\leq 0 \end{array}\right. \end{array}$$ (5)

where *d* and $\hat{d}$ are the decomposed and the thresholded coefficient respectively. The threshold, *τ*, is an estimated noise level, calculated by Median Absolute Deviation (MAD) [40, 41],

$$\begin{array}{c} \tau =\frac{\text{median}(|d^{*}|)}{0.6745} \end{array}$$ (6)

where d* is a vector of coefficients of the high frequency components. Afterwards, the high-frequency pattern was constructed using the thresholded coefficients (Fig 1D). The reconstructed high-spatial and low-spatial frequency patterns were represented in the space *θ* − *z*. The corresponding visualisation can be obtained by projecting these 2D patterns back to the tibia geometry in accordance with the coordinates of *θ* and *z*.

### Evaluation of the algorithmic accuracy

To evaluate the algorithmic accuracy, the proposed analysis framework was applied to datasets I and II. The datasets consisted of four different scenarios including manipulated global and local activity on a reference tibia bone surface. Each of the scenarios is described below:

#### Scenario 1: Zero low-spatial frequency and zero high-spatial frequency activity

**Fig 2:**
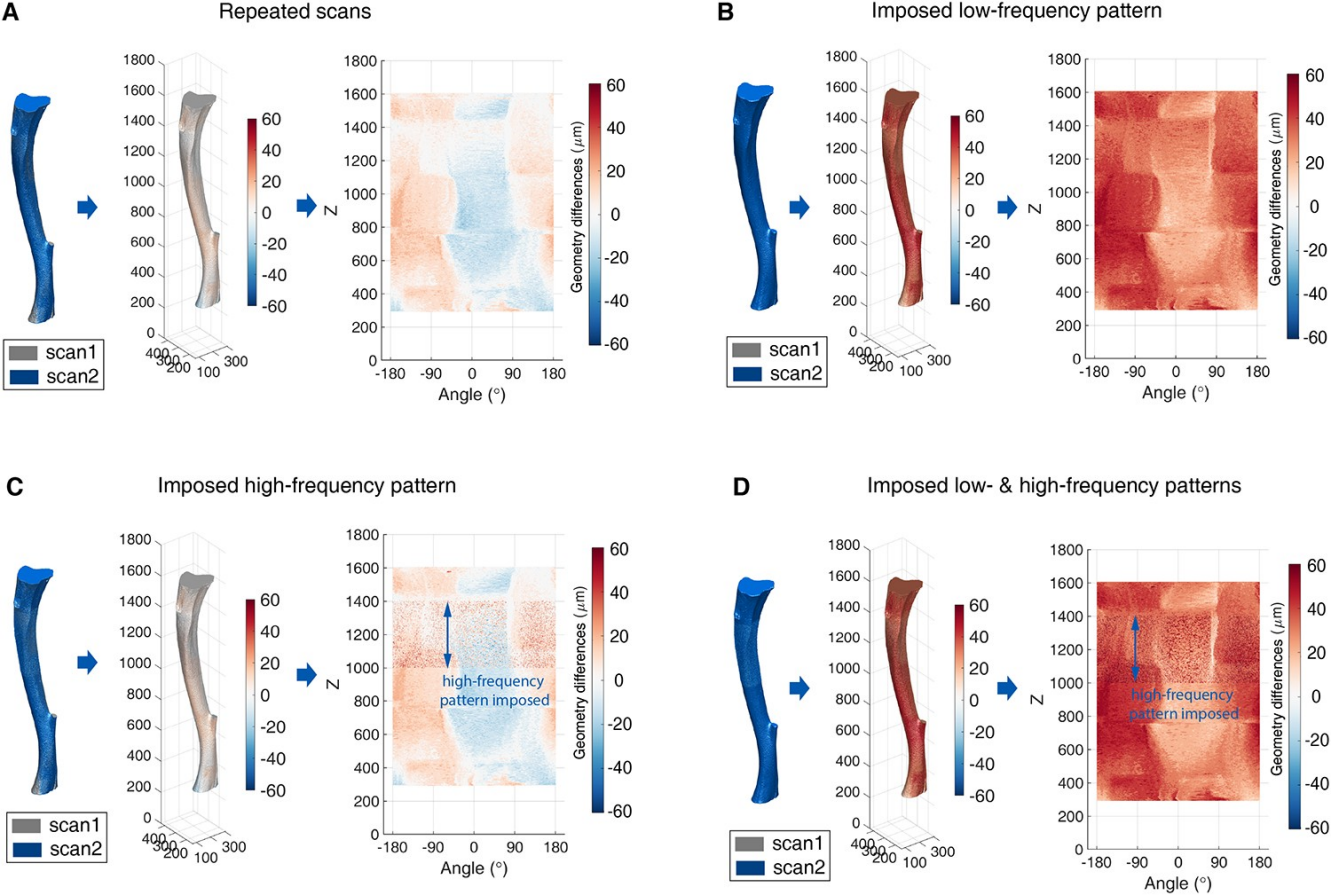
Prepared data for the algorithmic accuracy analysis. (A) Scenario 1: zero low- and high-spatial frequency activity; (B) Scenario 2: only imposed low-spatial frequency activity; (C) Scenario 3: only imposed high-spatial frequency activity; (D) Scenario 4: imposed low- and high-spatial frequency activity; In each figure, from left to right, they correspond to the geometry of periosteum at the first and the second scan at different scenario, the corresponding geometric changes and the unwrapped geometry change maps, respectively. The colourmap is used to indicate the magnitude and direction of the geometry difference, i.e., the warm colour (positive value) corresponds to the outward-pointing normal direction of the bone surface while the cold colour (negative value) corresponds to the inward-pointing normal direction.

The *in vivo* scans of the same tibia were collected one after the other (less than 190 minutes apart) to obtain repeated images of the same structures and including the typical noise in the acquired images. The corresponding level set function of these two scans were denoted as Φ_*t*_ and $\Phi_{t}^{*}$. The changes to the bone cells on the extracellular matrix were considered to be minimal in between the two measurements, Φ_*t*_ and $\Phi_{t}^{*}$, owing to their very short time duration. Therefore, the low-spatial (global) and high-spatial (local) frequency activities on the tibia surface should ideally be zero and the value estimated from the proposed framework would provide a quantitative assessment of possible systematic and random errors. The analysis was repeated on 5 specimens (a single example is reported in Fig 2A).

#### Scenario 2: Imposed low-spatial frequency activity

This synthetic bone surface was created by imposing a uniform growth on the entire periosteum, while no local changes were imposed. The same set of repeated *in vivo* scans with the Scenario 1 were used. The simulation of bone growth was carried out conveniently via the level-set function that was constructed in the first step of the framework. To be specific, the level-set functions of the periosteum at the consecutive scans were Φ_*t*_ and $\Phi_{t}^{*}$. Φ_*t*_ generating Φ_*t*+1_ directly does not review the influence of the random various that maybe presence in the real data. Hence, Φ_*t*+1_ was generated from the different initial scan $\Phi_{t}^{*}$. A growth field *F*_*g*_ = *c* × **1** was imposed by

$$\begin{array}{c} \Phi_{t+1}=\Phi_{t}^{*}+F_{g} \end{array}$$ (7)

where **1** is the matrix of ones of the same size as Φ_*t*_. This method is limited by the inability of the LSM to manipulate any growth that is smaller than 1 voxel. The experimental data given in Lu *et al*. [18] showed that the average cortical thickness could increase by approximately 30*μm* between weeks 14 and 22. Therefore, the constant growth rate here was set to *c* = 3*voxels* ≈ 30*μm*. According to Eq (1), the initial periosteum is obtained by setting Γ_*t*_ = {**Ω** ∣ Φ_*t*_ = 0} while the periosteum after growth was synthesised as Γ_*t*+1_ = {**Ω** ∣ Φ_*t*+1_ = 0} (Fig 2B). The multi-resolution framework was then applied to Γ_*t*+1_ and Γ_*t*_.

#### Scenario 3: Imposed high-spatial frequency activity

In this scenario, images were synthesised with local bone changes simulated at random sites within a specific region of bone height. A similar process of modifying Φ(*t*) was carried out as in Scenario 2 and designed as follows,

$$\begin{array}{c} \Phi_{t+1}=\Phi_{t}^{*}+F_{r} \end{array}$$ (8)

where

$$\begin{array}{c} F_{r}=[\begin{array}{ccc} 0_{2} & K & 0_{1} \end{array}{]}^{⊺} \end{array}$$ (9)

The local changes were chosen to take place at bone height region *z* ∈ [*l*_1_, *l*_2_] with magnitude less than *a*. Matrices $\Phi ,F_{r}\in R^{m\times n\times l}$ and the zero matrices are $0_{1}\in R^{m\times n\times (l_{1}-1)}$, $0_{2}\in R^{m\times n\times (l-l_{2})}$. The local activity, $K=(k_{ijz})\in R^{m\times n\times (l_{2}-l_{1}+1)}$, was constructed as

$$\begin{array}{c} k_{ijz}=U\left(-a,a\right)B\left(b\right) \end{array}$$ (10)

where

$$\begin{array}{c} B\left(b\right)=\{\begin{array}{cc} 0 & \text{if}\;U(0,1)\geq b\\ 1 & \text{else}\; \end{array} \end{array}$$ (11)

and U(-a,a) is a function that generates uniform distributed random numbers in the interval [−*a*, *a*] to introduce local geometry changes at random sites, while *b* ∈ [0, 1] is the fraction of the activated sites.

In order to check if the framework is able to separate the region with high-spatial frequency activity, the local changes were only imposed in the region of *z* ∈ [1000, 1400] (*voxel*). It is assumed that the geometry changes introduced by local activity were smaller than the average bone growth (i.e., *a* = 3 *voxels*) with activity rate *b* = 0.3, which is in accordance with the sum of mineralising and resorbing surface (*MS*/*BS* + *ES*/*BS* [*μm*^2^/*μm*^2^]) measured in the female C57Bl/6J mice between week 26 and 15 days later of age, as reported in Birkhold *et al*. [17]. The analysis framework was applied to the surface Γ_*t*_ = {**Ω** ∣ Φ_*t*_ = 0} and the surface imposed local activity Γ_*t*+1_ = {**Ω** ∣ Φ_*t*+1_ = 0} (Fig 2C).

#### Scenario 4: Imposed low- and high-spatial frequency activity

The final scenario, aimed at mimicking a realistic situation, imposed both global and local bone changes simultaneously. This was performed by integrating the synthesis processes of Scenario 2 and Scenario 3,

$$\Phi_{t+1}=\Phi_{t}^{*}+F_{g}+F_{r}.$$ (12)

The framework was applied to the surface Γ_*t*_ and the surface with low- and high-spatial frequency activity Γ_*t*+1_ = {**Ω** ∣ Φ_*t*+1_ = 0} (Fig 2D).

#### Error quantification

In the algorithmic accuracy analysis, the systematic errors were quantified using RMSD, defined by

$$\begin{array}{c} RMSD=\sqrt{\frac{1}{n}\sum_{i=1}^{n}{\left(x_{i}-{\hat{x}}_{i}\right)}^{2}} \end{array}$$ (13)

where *x*_*i*_ is the synthetically imposed low-/high-spatial frequency activity and ${\hat{x}}_{i}$ is the estimated low-/high-spatial frequency activity using the framework. In addition, the statistical distributions of the magnitudes of the estimation errors in the different spatial scales are presented for evaluation.

### *In vivo* scans of wild type mouse tibia

To analyse the outcome of the proposed method on a real dataset, the analysis framework was applied to the *in vivo μ*CT scan of the right tibia of wild-type mice (dataset III). The spatio-temporal variation in low- and high-spatial frequency activity were quantified by averaging the measurements across the directions of *z* and *θ* separately. High-spatial frequency activity will automatically remove effects of bone formation and bone resorption, leaving this information to be zero mean, with high frequency information contained in positive and negative changes from the low frequency mean values. Since the information on the high-spatial frequency bone adaptation activity are contained in the positive and negative values of bone change, the integration of absolute values were used to quantify this activity. In addition, an activation rate, *r*, was used to measure the percentage of the bone surface where the local activity took place,

$$\begin{array}{c} r=\frac{A_{r}}{A_{s}} \end{array}$$ (14)

where *A*_*s*_ is the area of the bone surface and *A*_*r*_ is the area of the bone sites with local activities.

Data collected in this study are accessible at https://doi.org/10.15131/shef.data.8319956.

## Results

### Evaluation of the algorithmic accuracy

The results of applying the framework to the repeated *in vivo μ*CT scans with synthetically added global and/or local activities are provided for each of the four scenarios. They are presented also spatially to highlight the spatial location of the errors.

#### Scenario 1: Zero low-spatial frequency and zero high-spatial frequency activity

**Fig 3:**
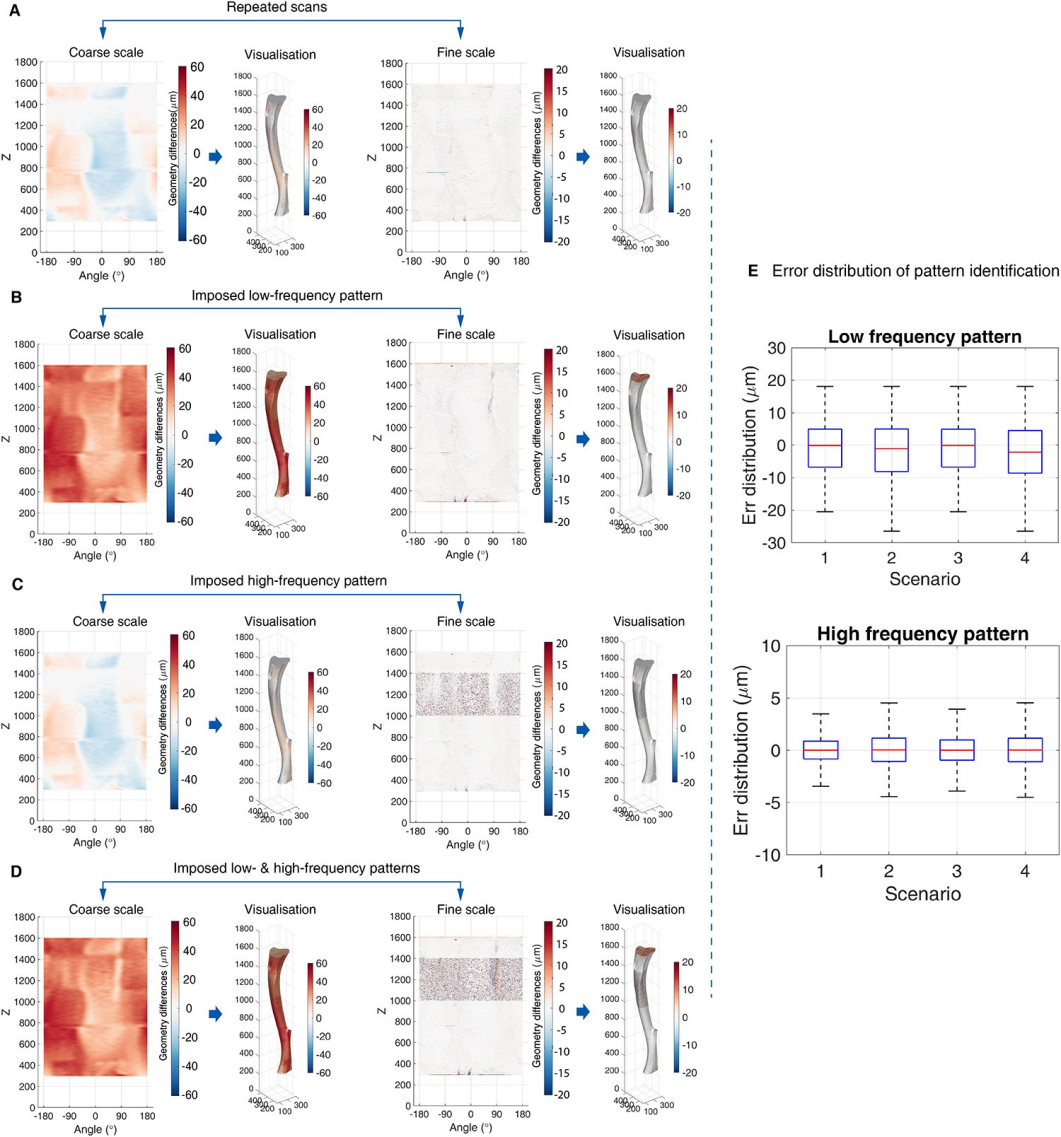
Evaluation of the algorithmic accuracy. (A) Multi-resolution analysis of the Scenario 1: zero low- and hight-spatial frequency activity. (B) Analysis of the Scenario 2: only imposed low-spatial frequency activity. (C) Analysis of the Scenario 3: only imposed high-spatial frequency activity. (D) Analysis of the Scenario 4: imposed low- and high-spatial frequency activity. In each figure, from left to right, they show the decomposed coarse scale of geometric changes at different scenarios, the corresponding visualisation, the fine scale patterns and the corresponding visualisation. (E) The box plot of error distributions at different scenarios. The red line in the middle indicates the median value, the top and bottom of the box are the 75% and 25% of the error distribution and the whiskers extend to the minimum and maximum values. In sub-figures (A-D), the colourmap is used to indicate the magnitude and direction of the geometric changes. The warm colour (positive value) corresponds to the outward-pointing normal direction of the bone surface while the cold colour (negative value) corresponds to the inward-pointing normal direction.

In this scenario, the obtained coarse and fine scale patterns after the multi-resolution analysis should reveal the errors in the estimation of the low- and high-spatial frequency patterns (Fig 3A). These errors can be introduced by the application of the framework as well as the pre-processing artifacts. For example, the alternating red and blue regions at coarse scale may indicate the translation errors generated in the 3D image registration (Fig 3A). Larger errors are observed in the identification of low-spatial frequency pattern in comparison with the identification of the high-spatial frequency pattern, evidenced by the error distribution of low-spatial frequency identification *ϵ* ∈ [−20*μm*, 20*μm*] and hight-spatial frequency identification *ϵ* ∈ [−5*μm*, 5*μm*] (Fig 3E). Similarly, the statistical analysis shows that the errors in low frequency activity identification was about five times as much as the errors in high frequency activity identification, evidenced on both the periosteum and the endosteum (Table 1, RMSD at periosteum: 11.6*μm* (low frequency activity) vs. 2.3*μm* (high frequency activity); Endosteum: 10.9*μm* vs. 2.1*μm*).

**Table 1:** Systematic errors in different scenarios (RMSD, N = 5 mouse).

		Scenario 1	Scenario 2	Scenario 3	Scenario 4
Errors of low-spatial frequency pattern identification (Mean ± SD)	Periosteum (*μm*)	11.6 ± 3.2	12.1 ± 3.2	11.5 ± 3.0	12.3 ± 3.1
	Endosteum (*μm*)	10.9 ± 2.5	11.2 ± 2.5	10.9 ± 2.5	11.2 ± 2.5
Errors of high-spatial frequency pattern identification (Mean ± SD)	Periosteum (*μm*)	2.3 ± 0.6	6.5 ± 0.2	6.7 ± 0.4	7.9 ± 0.2
	Endosteum (*μm*)	2.1 ± 0.4	6.3 ± 0.2	7.2 ± 0.2	7.8 ± 0.2

#### Scenario 2: Imposed low-spatial frequency activity

The framework was applied to the repeated scans with synthetically added uniform global bone growth (Fig 2B). In this scenario, the decomposed pattern at coarse scale should correspond to the approximate global bone growth while the fine scale would reveal the errors incurred in the high-spatial frequency activity identification. The results are shown in Fig 3B. The decomposed coarse pattern has largely captured the imposed constant growth on periosteum subject to minor fluctuations similarly to the previous scenario (Fig 3B and 3A). The errors in the low-spatial frequency activity identification were quantified by subtracting the imposed growth from the coarse scale pattern and were observed mainly to be in the range of [−20*μm*, 20*μm*] (Fig 3E). Meanwhile, additional variations were introduced in the identification of high-frequency activity compared to the Scenario 1, as evidenced by the larger errors distribution (Fig 3E). These errors can be attributed to the joint effect of low-frequency activity estimation errors influencing high-frequency activity estimation. However, the errors were still much smaller than those related to the identification of the low-frequency activity with RMSD at the periosteum being equal to 12.1*μm* (low frequency pattern) and 6.4*μm* (high frequency pattern) and RMSD at the endosteum being equal to 11.2*μm* (low frequency pattern) and 6.3*μm* (high frequency pattern) (Table 1).

#### Scenario 3: Imposed high-spatial frequency activity

The obtained coarse and fine patterns were expected to separately reveal the errors of bone low-spatial frequency activity identification and high-spatial frequency patterns. The errors associated with the coarse patterns were consistent with the error pattern observed in Scenario 1 (Fig 3C and 3A). The pattern at the fine scale showed higher occurrence of local geometry changes at the bone surface of the proximal metaphysis (*z* ∈ [1000, 1400], Fig 3C), where the local geometry changes were imposed (Fig 2C). The quantified errors in Table 1 indicate that the high-spatial frequency activity identification was less accurate compared with the first scenario with RMSD at periosteum equals 2.3*μm* (Scenario 1) 6.7*μm* (Scenario 3); Endosteum: 2.1*μm* (Scenario 1) vs. 7.2*μm* (Scenario 3)). However, the increased errors were still not comparable to the errors existing in the low-spatial frequency pattern identification (RMSD at periosteum: 11.5*μm* (low frequency activity) vs. 6.7*μm* (high frequency activity); Endosteum: 10.9*μm* vs. 7.2*μm*, Table 1).

#### Scenario 4: Imposed low- and high-spatial frequency activity

The analysis results of this scenario is shown in Fig 3D. A visual inspection appears to show that the framework can separate the global changes and the irregular local changes at the region where the high frequency activity was imposed. The coarse pattern identified the imposed 30*μm* growth over the space while the fine scale pattern captured the region pertaining to the height interval [1000, 1400] where the extra high-spatial frequency activity was imposed (Fig 3D). The errors were then quantified by subtracting the imposed global and local activity from these approximated patterns. Compared to the Scenario 1, higher errors were introduced in the identification of both bone low- and high-spatial frequency patterns. Additionally, the RMSD values in Table 1 show that the errors of high-spatial frequency activity identification were smaller in comparison to those related to the low frequency activity with RMSD at the periosteum was 12.3*μm* (low-frequency pattern) and 7.9*μm* (high-frequency pattern), and RMSD at the endosteum was 11.2*μm* (low-frequency pattern) and 7.8*μm* (high-frequency pattern)(Table 1).

### *In vivo* scans of wild-type mouse tibiae

**Fig 4:**
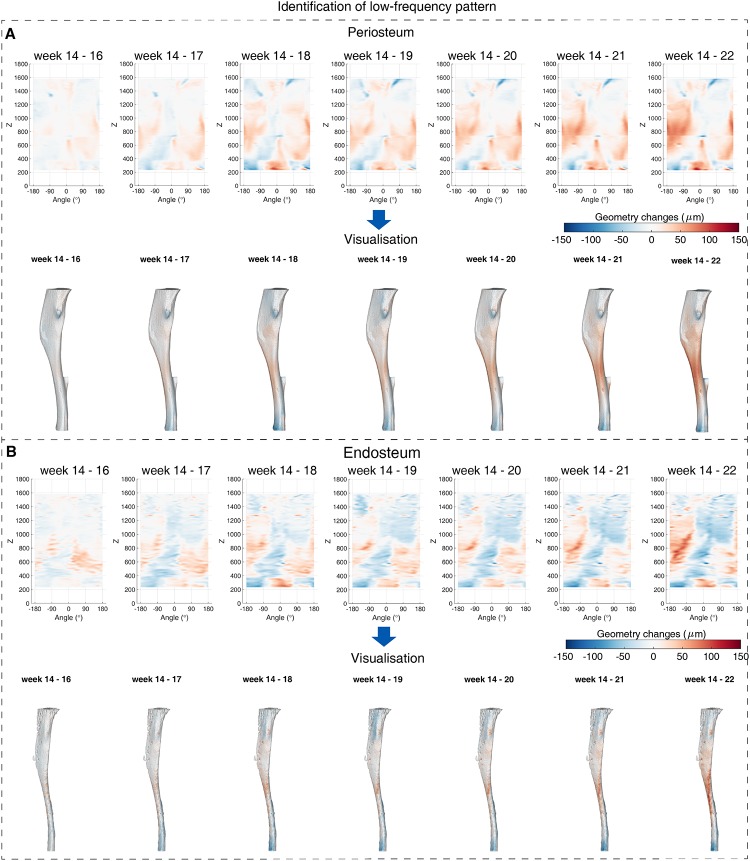
Identified bone low-spatial frequency activity from week 14 to week 22 of a wild-type mouse tibia. (A) The identified low frequency activity on periosteum (Top) and the corresponding visualisation (Bottom). (B) The identified low frequency patterns on endosteum (Top) and the corresponding visualisation (Bottom). In sub-figures, the patterns from left to right correspond to the geometric changes from week 14 to week 22.

**Fig 5:**
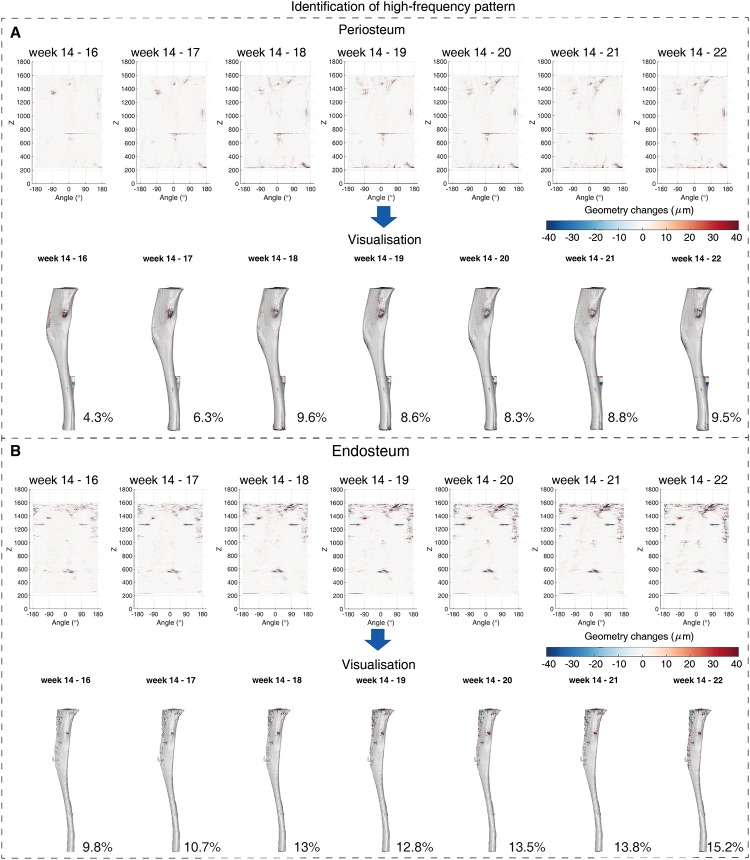
Identified high-spatial frequency activity at bone surface from week 14 to week 22 of a wild-type mouse tibia. (A) The identified hight frequency activity patterns on periosteum (top) and the corresponding visualisation (bottom). The activation rate is presented at the bottom right. (B) The identified high frequency activity patterns on endosteum (top) and the corresponding visualisation (bottom) with the activation rate at the bottom right. In sub-figures, the patterns from left to right correspond to the geometric changes from week 14 to 22.

The proposed framework provides an approach to quantify the bone low- and high-spatial frequency activities over time, not just over space. This spatiotemporal analysis of bone geometry change patterns of one wild-type mouse tibia over 8 weeks is illustrated in Figs 4 and 5. The identified low-spatial frequency patterns show that the bone formation was observed over most of the region at periosteum and with increasing time, the formation was observed to increase in magnitude and over space (Fig 4A). Compared to periosteum, bone resorption was observed over larger regions on endosteal surface (Fig 4B).

Comparing the low-spatial frequency patterns from week 14 to week 22, the high-spatial frequency patterns were more spatially disconnected and the magnitudes were smaller. The geometry changes contributed by low-spatial frequency activity were estimated to be in the range of [−150*μm*, 150*μm*] whereas the changes contributed by high frequency activity were in the range of [−40*μm*, 40*μm*] (Figs 4A vs. 5A and 4B vs. 5B). The high-spatial frequency activities that can either be bone formation or resorption were mainly activated at the tibia proximal end as well as the anterior border. When comparing the estimation at the same week spatially, the endosteal surface was identified as having more activated sites compared to the periosteal surface, and especially concentrated in the most proximal part (Fig 5A and 5B). Over time, more sites were estimated as being activated until week 18 when the activation rate becomes relatively stable, which was observed on both periosteal and endosteal surfaces (Fig 5A and 5B).

**Fig 6:**
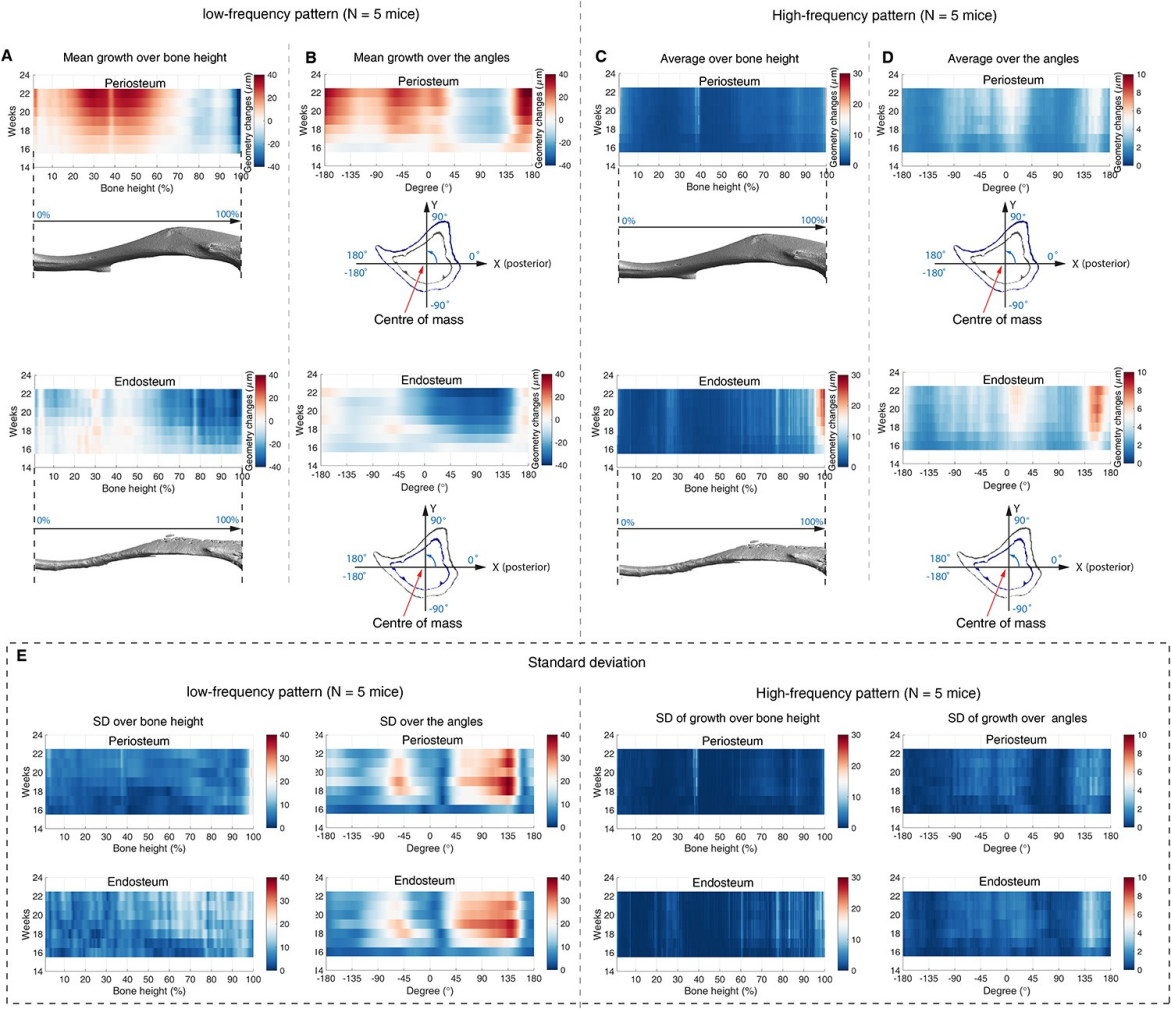
Spatial-temporal analysis of bone geometry changes of a wild-type mouse tibia. (A-B) The spatially averaged low-spatial frequency activity patterns from week 14 to 22. (C-D) The spatially averaged high-spatial frequency activity patterns from week 14 to 22. The averaged pattern over bone height are shown in sub-figures A and C (top: periosteum, bottom: endosteum). The averaged pattern over angles are shown in sub-figures B and D (top: periosteum, bottom: endosteum). (E) The corresponding SD maps of the sub-figures (A-D)(N = 5 mice).

The averaged growth over bone height and direction (angles) across time are shown in Fig 6A and the corresponding Standard Deviation (SD) are given in Fig 6E (*N* = 5 mice). It reveals that the bone formation area initialised at the mid-shaft of the cortical bone, and started to expand spatially. At the end of the week 22, the bone formation region represented over 70% of the bone height, and on both surfaces (Fig 6A). The averaged bone growth over angles, *θ*, did not present a clear growth tendency over time (Fig 6B).

The mean geometry changes due to the high-spatial frequency patterns over bone height/angles and time are showed in Fig 6C and the corresponding SD are given in Fig 6D (*N* = 5 mice). The variation across the bone height showed that the activity was highest at the proximal ends of the tibia while the endosteum showed more activated sites than the periosteum (Fig 6C). The variation across the angle did not show a clear tendency over time (Fig 6D).

## Discussion

In this study, a novel framework was proposed for separating the contribution of low- and high-spatial frequency activity to bone geometry changes in the cortical bone. The framework was applied to analyse the cortical surface changes over the 80% of tibia length. It should be noted that only the geometry changes at convex closed surfaces (periosteum and endosteum) were considered. The application of LSM enabled the tracking of the complicated topology changes at bone surfaces (periosteum and endosteum). The multi-resolution analysis estimated the activity patterns at the bone surface according to their spatial frequency characteristics. The combination of these two steps provided a tool to spatio-temporally quantify and visualise the low- and high-spatial frequency activity from the *μ*CT scans of mouse tibia. The accuracy of the framework was analysed through scenarios using repeated scans with synthetically manipulated global and local geometry changes. Under these synthetic test conditions, the RMSD was approximately 15*μm* for low frequency activity estimation and 7*μm* for the high-spatial frequency estimation. The largest errors occurred in the scenarios that were imposed with both global and local activity. The proposed framework was then applied for the quantification of bone adaptations at periosteum and endosteum of a set of wild-type mouse tibiae. The quantified level of uncertainties provides a benchmark for the degree of accuracy that may be expected from analysis of real bone *μ*CT scans.

The analysis of the accuracy of the proposed method showed that the errors increased with the complexity of the synthetic bone adaptation scenarios. Analysis of the two repeated scans on the same day (nominally zero low- and high-spatial frequency changes) were limited to a single voxel. The errors across all four scenarios were approximately 1.5 voxels and 0.7 voxels for low- and high-spatial frequency activity estimation, respectively. These errors can be attributed by three sources of uncertainties. Firstly, the rigid registration process ignores the non-rigid transformation of bone adaptation and lead to spatial errors in the analysis. This will lead to low-spatial frequency errors in the analysis. Secondly, the synthetically created bone geometry in scenarios 2-4 (imposed high- and/or low-spatial frequency changes) introduces quantisation errors due to the fact that the LSM was used for segmentation and creation of a continuous surface, which is then quantised after the addition of bone adaption. Such errors have more propensity to affect high-spatial frequency changes. The third source of error arises from the measurement noise, which can appear inseparable from the high spatial frequency effects. The idea of denoising through sparsification is applied to reduce the effects of this source of error.

The spatio-temporal analysis of the *μ*CT image sequences of the right mouse tibia confirmed the expectation and observations that the estimated bone activity increased over time. The presence of more high-spatial frequency activity regions on proximally than distal growth plate is probably due to the presence of large portion of trabecular bone in that area, which is more metabolically active compared to cortical bone. We need to acknowledge that this approach does not capture potential differences in local mineralization, which could happen in the cortical bone of the mouse tibia, but only localized changes of geometry due to resorption cavities at high-spatial frequency.

Though the paper has proposed a novel approach to analysing bone geometry changes, the study has a number of limitations. By using synthetic benchmarks in the approach used to quantify the accuracy of the proposed algorithm, the realism was sacrificed in the need for controllability and reliability. It may be useful to complement this study with a sensitivity analysis that explores how the quality of the *μ*CT images (e.g., their signal-to-noise-ratio) affects the accuracy of the proposed algorithm. Secondly, in order to set a regular array for the following multi-resolution analysis, the nonlinear projection applied to unwrap the 3D geometry changes may require compensation. Introducing the variable of radius from mass centre to bone surface can improve the accuracy of projection, which however requires an indirect way to construct a regular array. Finally, the separation of bone geometry changes in different spatial scales does not directly provide the information concerning bone growth and remodelling, the real quantities of interest in such analysis.

The proposed algorithm estimated bone activities at different spatial frequency scales. Potentially, this can be a solution to a long-standing problem in the evaluation of bone adaptation to interventions using murine models [15, 16]. The *in vivo μ*CT measurements do not separate between bone growth and bone adaptation; while the use of older mice does reduce the problem [11], it does not remove it entirely. This is not only a methodological detail: in most cases these murine studies are models of what occur in post-menopausal women, where clearly no skeletal growth is observed. It is important therefore, for example when investigating anabolic drugs, to separate their effect on the growth metabolism, which is not relevant for adult humans, from that on bone remodelling, which is instead very relevant for ageing humans. Specifically, both longitudinal growth (at the growth plates) and appositional growth (at the periosteal and endosteal surfaces) occur simultaneously over large portions of the bone surface. Thus, the global geometry changes are associated with low-spatial frequency characteristics [29, 30]. Tibial growth in the mice occurs at the periosteum, endosteum and the growth plates. Longitudinal growth at the growth plates occurs quite uniformly, and while it is possible that the rate of linear growth is affected by mechanical loading, there are no histological evidence of mechano-regulated bone resorption just below the growth plates. Thus, we can safely assume that all changes in length are exclusively due to growth and not to remodelling. Bone length was measured from the pixel of the most tibial bone to the most distal bone. To account for the longitudinal growth (small in the considered age range, approximately 300 − 400 *μ*m [42]). We have considered 80% of the tibia length below the proximal growth plate. The corresponding growth at periosteum and endosteum then can be estimated in the proposed framework through the normal distance between the bone surfaces at different time using level-set method and assuming that low-spatial frequency changes are associated to growth. In terms to the bone remodelling, it is a dynamic process involving both bone resorption and formation, which occur locally at the bone surface [29, 30]. Therefore, changes in bone geometry due to the bone remodelling can be hypothesised to have high-spatial frequency characteristics. To date, the lack of experimental validation is the major limitation. Though it is possible to compare the proposed algorithm with bone histomorphometry study about bone remodelling [6], such a study could be done only in small portions of the tibia biopsy, and is not suitable as the reference method for validation.

In summary, the proposed framework analysed bone geometry changes from the *in vivo μ*CT scans, and performed the spatio-temporal quantification and visualisation of these changes at different spatial scales. This analysis framework can be a useful tool to the longitudinal studies based on *in vivo μ*CT data.

## Supporting information

S1 Fig Hausdorff distance between the reconstructed bone surfaces using LSM and the surfaces using marching cubes. (a) A visualisation of the spatial distribution of the Hausdorff distance. (b) The normalised histogram of the Hausdorff distance, which is obtained from 40 *in vivo μ*CT scans of wild type mouse tibiae. The Supplementary S2, S4, S6 and S8 Figs show the low-spatial frequency patterns of *in vivo* scans of four wild-type mouse tibiae, which are different from the one provided in Fig 4. The corresponding high-spatial frequency patterns are shown in S3, S5, S7 and S9 Figs.
(PDF)

S2 Fig Identified bone low-spatial frequency activity from week 14 to week 22 of a wild-type mouse tibia. (A) The identified low frequency activity on periosteum (Top) and the corresponding visualisation (Bottom). (B) The identified low frequency patterns on endosteum (Top) and the corresponding visualisation (Bottom). In sub-figures, the patterns from left to right correspond to the geometric changes from week 14 to week 22.
(PDF)

S3 Fig Identified high-spatial frequency activity at bone surface from week 14 to week 22 of a wild-type mouse tibia. (A) The identified hight frequency activity patterns on periosteum (top) and the corresponding visualisation (bottom). The activation rate is presented at the bottom right. (B) The identified high frequency activity patterns on endosteum (top) and the corresponding visualisation (bottom) with the activation rate at the bottom right. In sub-figures, the patterns from left to right correspond to the geometric changes from week 14 to 22.
(PDF)

S4 Fig Identified bone low-spatial frequency activity from week 14 to week 22 of a wild-type mouse tibia. (A) The identified low frequency activity on periosteum (Top) and the corresponding visualisation (Bottom). (B) The identified low frequency patterns on endosteum (Top) and the corresponding visualisation (Bottom). In sub-figures, the patterns from left to right correspond to the geometric changes from week 14 to week 22.
(PDF)

S5 Fig Identified high-spatial frequency activity at bone surface from week 14 to week 22 of a wild-type mouse tibia. (A) The identified hight frequency activity patterns on periosteum (top) and the corresponding visualisation (bottom). The activation rate is presented at the bottom right. (B) The identified high frequency activity patterns on endosteum (top) and the corresponding visualisation (bottom) with the activation rate at the bottom right. In sub-figures, the patterns from left to right correspond to the geometric changes from week 14 to 22.
(PDF)

S6 Fig Identified bone low-spatial frequency activity from week 14 to week 22 of a wild-type mouse tibia. (A) The identified low frequency activity on periosteum (Top) and the corresponding visualisation (Bottom). (B) The identified low frequency patterns on endosteum (Top) and the corresponding visualisation (Bottom). In sub-figures, the patterns from left to right correspond to the geometric changes from week 14 to week 22.
(PDF)

S7 Fig Identified high-spatial frequency activity at bone surface from week 14 to week 22 of a wild-type mouse tibia. (A) The identified hight frequency activity patterns on periosteum (top) and the corresponding visualisation (bottom). The activation rate is presented at the bottom right. (B) The identified high frequency activity patterns on endosteum (top) and the corresponding visualisation (bottom) with the activation rate at the bottom right. In sub-figures, the patterns from left to right correspond to the geometric changes from week 14 to 22.
(PDF)

S8 Fig Identified bone low-spatial frequency activity from week 14 to week 22 of a wild-type mouse tibia. (A) The identified low frequency activity on periosteum (Top) and the corresponding visualisation (Bottom). (B) The identified low frequency patterns on endosteum (Top) and the corresponding visualisation (Bottom). In sub-figures, the patterns from left to right correspond to the geometric changes from week 14 to week 22.
(PDF)

S9 Fig Identified high-spatial frequency activity at bone surface from week 14 to week 22 of a wild-type mouse tibia. (A) The identified hight frequency activity patterns on periosteum (top) and the corresponding visualisation (bottom). The activation rate is presented at the bottom right. (B) The identified high frequency activity patterns on endosteum (top) and the corresponding visualisation (bottom) with the activation rate at the bottom right. In sub-figures, the patterns from left to right correspond to the geometric changes from week 14 to 22.
(PDF)
