# Supplementary material for: A new method to monitor bone geometry changes at different spatial scales in the longitudinal in vivo μCT studies of mice bones
Source: PLoS One. 2019 Jul 22;14(7):e0219404. doi: 10.1371/journal.pone.0219404 (PMC6645529; doi:10.1371/journal.pone.0219404)
Supplement: S1 Fig — (a) A visualisation of the spatial distribution of the Hausdorff distance. (b) The normalised histogram of the Hausdorff distance, which is obtained from 40 in vivo μCT scans of wild type mouse tibiae. The Supplementary S2, S4, S6 and S8 Figs show the low-spatial frequency patterns of in vivo scans of four wild-type mouse tibiae, which are different from the one provided in Fig 4. The corresponding high-spatial frequency patterns are shown in S3, S5, S7 and S9 Figs. (PDF) [file pone.0219404.s001.pdf]

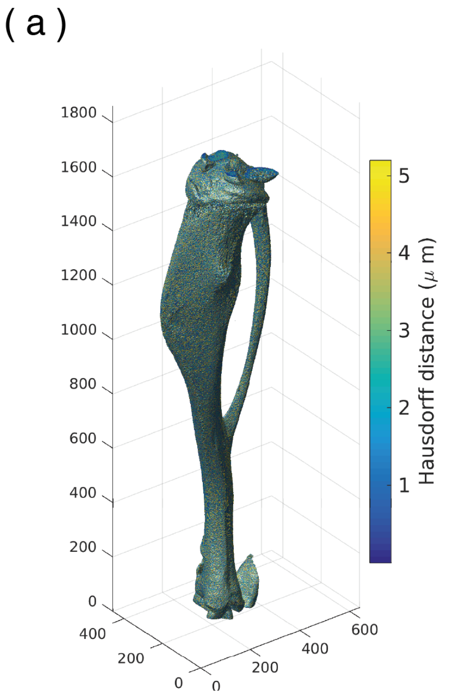

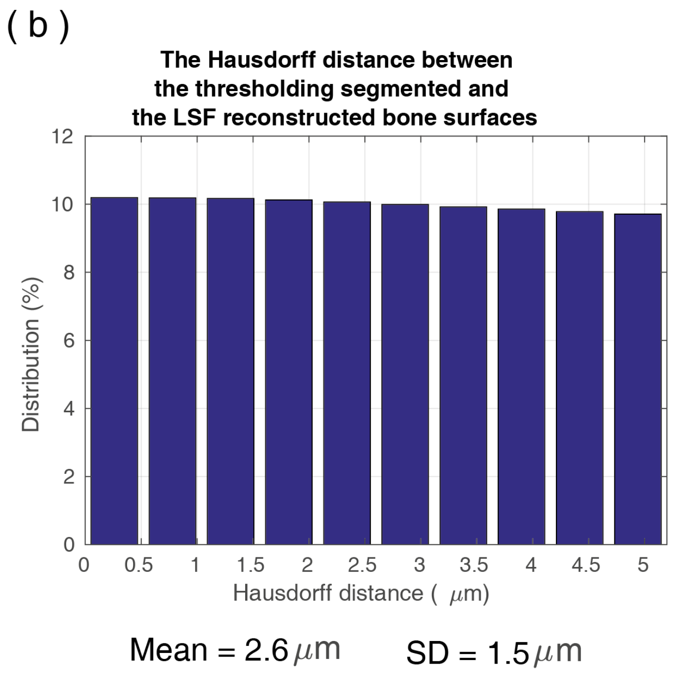

Figure S1: **Hausdorff distance between the reconstructed bone surfaces using level-set method (LSM) and the surfaces using marching cubes.** (a) A visualisation of the spatial distribution of the Hausdorff distance. (b) The normalised histogram of the Hausdorff distance, which is obtained from 40 *in vivo* μCT scans of wild type mouse tibiae.
