# Supplementary material for: A new method to monitor bone geometry changes at different spatial scales in the longitudinal in vivo μCT studies of mice bones
Source: PLoS One. 2019 Jul 22;14(7):e0219404. doi: 10.1371/journal.pone.0219404 (PMC6645529; doi:10.1371/journal.pone.0219404)
Supplement: S5 Fig — (A) The identified hight frequency activity patterns on periosteum (top) and the corresponding visualisation (bottom). The activation rate is presented at the bottom right. (B) The identified high frequency activity patterns on endosteum (top) and the corresponding visualisation (bottom) with the activation rate at the bottom right. In sub-figures, the patterns from left to right correspond to the geometric changes from week 14 to 22. (PDF) [file pone.0219404.s005.pdf]

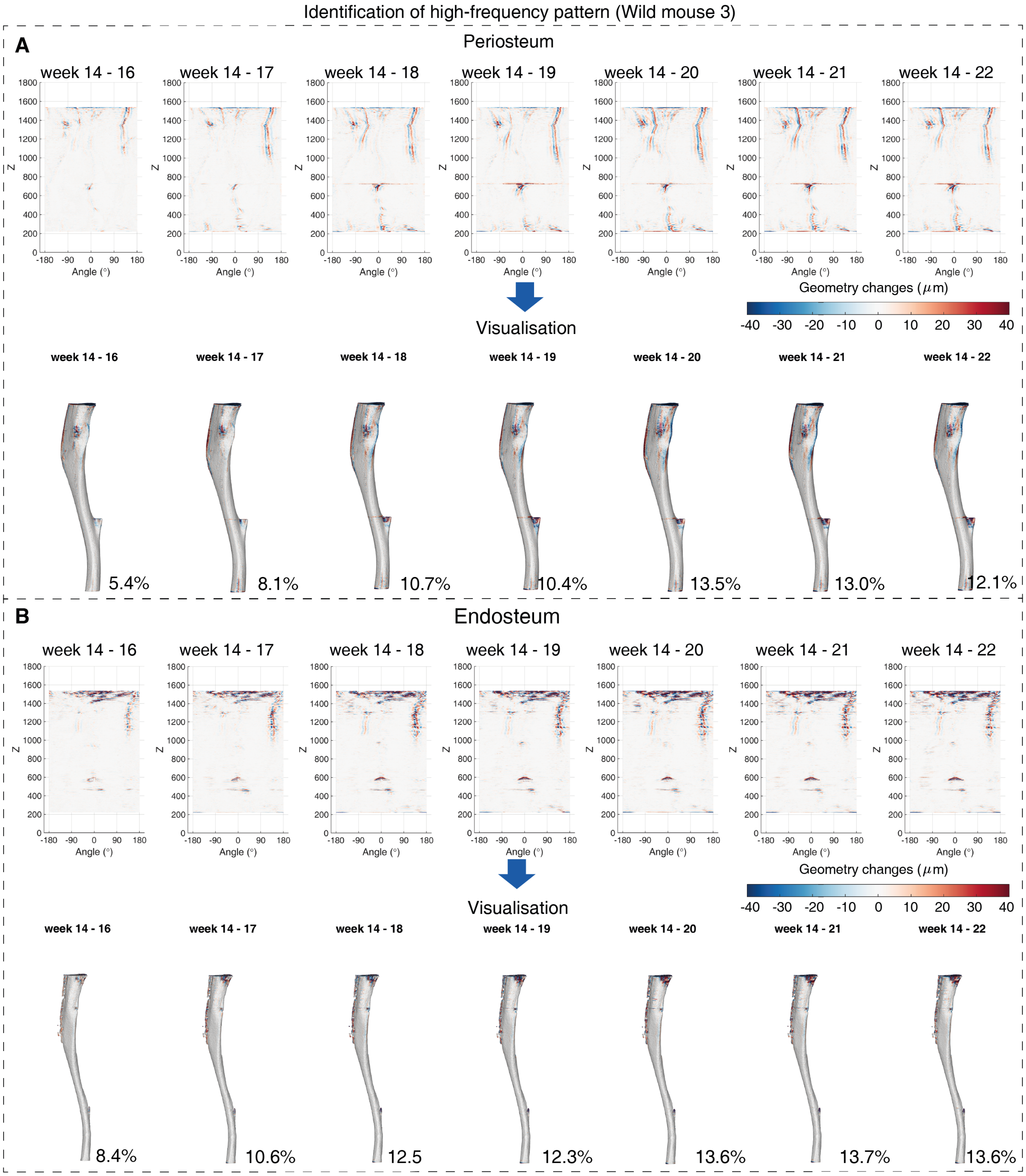

Figure S5: **Identified high-spatial frequency activity at bone surface from week 14 to week 22 of a wild-type mouse tibia.** (A) The identified hight frequency activity patterns on periosteum (top) and the corresponding visualisation (bottom). The activation rate is presented at the bottom right. (B) The identified high frequency activity patterns on endosteum (top) and the corresponding visualisation (bottom) with the activation rate at the bottom right. In sub-figures, the patterns from left to right correspond to the geometric changes from week 14 to 22.
