# Supplementary material for: A new method to monitor bone geometry changes at different spatial scales in the longitudinal in vivo μCT studies of mice bones
Source: PLoS One. 2019 Jul 22;14(7):e0219404. doi: 10.1371/journal.pone.0219404 (PMC6645529; doi:10.1371/journal.pone.0219404)
Supplement: S8 Fig — (A) The identified low frequency activity on periosteum (Top) and the corresponding visualisation (Bottom). (B) The identified low frequency patterns on endosteum (Top) and the corresponding visualisation (Bottom). In sub-figures, the patterns from left to right correspond to the geometric changes from week 14 to week 22. (PDF) [file pone.0219404.s008.pdf]

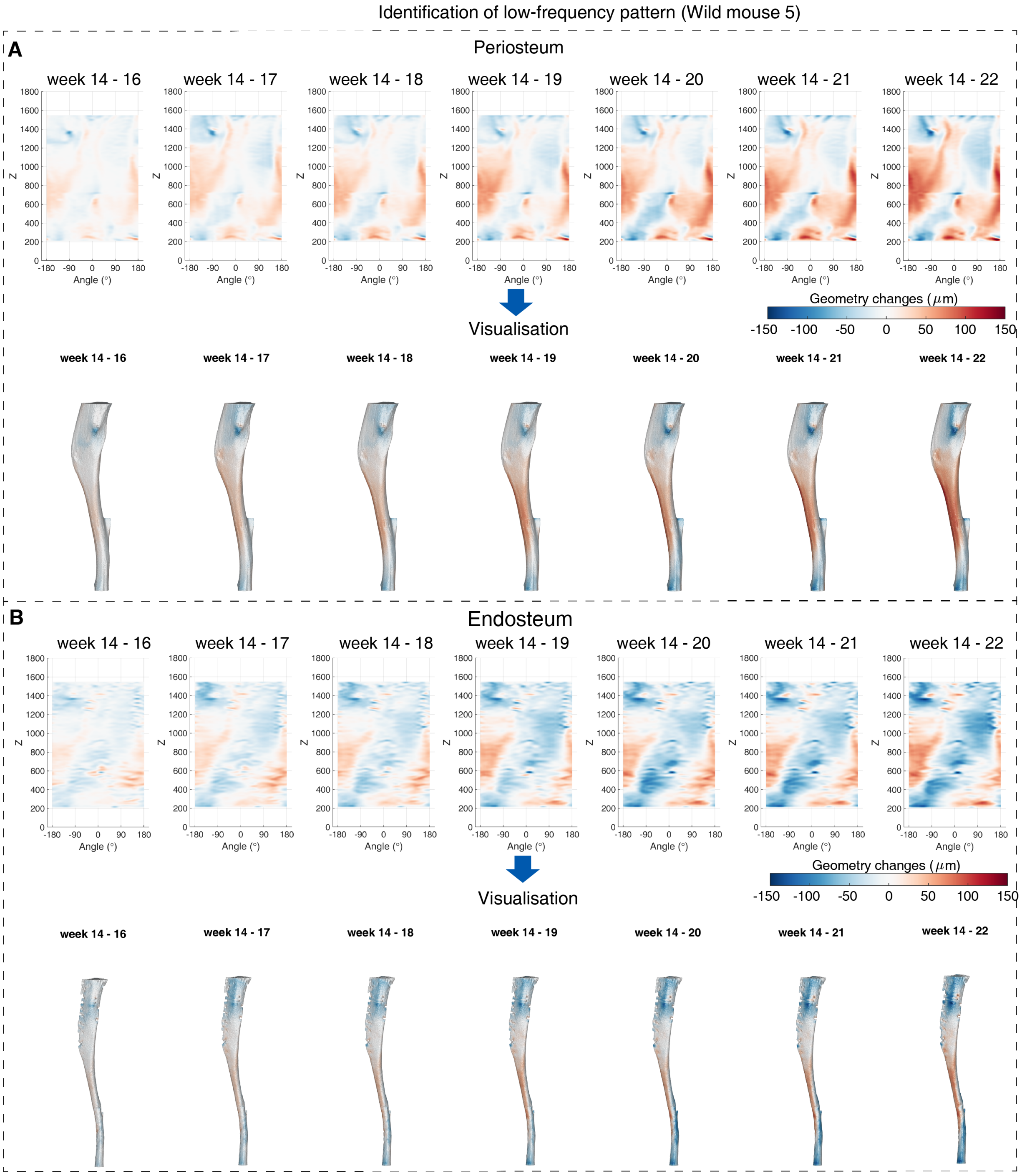

Figure S8: **Identified bone low-spatial frequency activity from week 14 to week 22 of a wild-type mouse tibia.** (A) The identified low frequency activity on periosteum (Top) and the corresponding visualisation (Bottom). (B) The identified low frequency patterns on endosteum (Top) and the corresponding visualisation (Bottom). In sub-figures, the patterns from left to right correspond to the geometric changes from week 14 to week 22.
